# Perturbation-Response Scanning Reveals Ligand Entry-Exit Mechanisms of Ferric Binding Protein

**DOI:** 10.1371/journal.pcbi.1000544

**Published:** 2009-10-23

**Authors:** Canan Atilgan, Ali Rana Atilgan

**Affiliations:** Faculty of Engineering and Natural Sciences, Sabanci University, Istanbul, Turkey; National Cancer Institute, United States of America and Tel Aviv University, Israel

## Abstract

We study apo and holo forms of the bacterial ferric binding protein (FBP) which exhibits the so-called ferric transport dilemma: it uptakes iron from the host with remarkable affinity, yet releases it with ease in the cytoplasm for subsequent use. The observations fit the “conformational selection” model whereby the existence of a weakly populated, higher energy conformation that is stabilized in the presence of the ligand is proposed. We introduce a new tool that we term perturbation-response scanning (PRS) for the analysis of remote control strategies utilized. The approach relies on the systematic use of computational perturbation/response techniques based on linear response theory, by sequentially applying directed forces on single-residues along the chain and recording the resulting relative changes in the residue coordinates. We further obtain closed-form expressions for the magnitude and the directionality of the response. Using PRS, we study the ligand release mechanisms of FBP and support the findings by molecular dynamics simulations. We find that the residue-by-residue displacements between the apo and the holo forms, as determined from the X-ray structures, are faithfully reproduced by perturbations applied on the majority of the residues of the apo form. However, once the stabilizing ligand (Fe) is integrated to the system in holo FBP, perturbing only a few select residues successfully reproduces the experimental displacements. Thus, iron uptake by FBP is a favored process in the fluctuating environment of the protein, whereas iron release is controlled by mechanisms including chelation and allostery. The directional analysis that we implement in the PRS methodology implicates the latter mechanism by leading to a few distant, charged, and exposed loop residues. Upon perturbing these, irrespective of the direction of the operating forces, we find that the cap residues involved in iron release are made to operate coherently, facilitating release of the ion.

## Author Summary

Upon binding ligands, many proteins undergo structural changes compared to the unbound form. We introduce a methodology to monitor these changes and to study which mechanisms arrange conformational shifts between the liganded and free forms. Our method is simple, yet it efficiently characterizes the response of proteins to a given perturbation on systematically selected residues. The coherent responses predicted are validated by molecular dynamics simulations. The results indicate that the iron uptake by the ferric binding protein is favorable in a thermally fluctuating environment, while release of iron is allosterically moderated. Since ferric binding protein exhibits a high sequence identity with human transferrin whose allosteric anion binding sites generate large conformational changes around the binding region, we suggest mutational studies on remotely controlling sites identified in this work.

## Introduction

Functional proteins are complex structures, which may remain mainly unmodified as a result of a multitude of mutations [1], yet may have their energy surface go through significant changes upon perturbing highly specific regions [2]–[4]. The various accessible states populated may be manipulated by inducing short and long-range conformational changes in the structure [5]; alternatively, a dynamical control may take place without any significant structural variation [6],[7]. To explore the presence or the absence of such “shifts in the energy landscapes,” [8] one needs to perturb the protein structure, and observe the response [9]. The perturbation may be in the form of changing the environmental factors (e.g. changes in ionic concentration [10]), or may target specific locations on the structure itself, either through chemically modifying the residues (inserting mutations) [11] or by inducing site-specific perturbations (e.g. as is done in single molecule experiments [12], or through ligand binding). Ubiquitous post-translational modifications are also possible. The response may be measured directly, as a change in the overall conformation of the protein [13], or indirectly, e.g., through determining the kinetic parameters, and proposing kinetic models that explain the observations.[14],[15] The purpose in such work is to understand and therefore control the response of the protein for a plethora of reasons, including, but not limited to, the design of efficient drugs [16],[17], or to tailor enzymes serving as “materials.”[18]

Linear response theory (LRT) has been recently used to study conformational changes undergone by proteins under selected external perturbations [19]. This approach has recently been applied to the study of the conformational switching upon phosphorylation [20]. In this study, we develop a toolkit that we term perturbation-response scanning (PRS) which is based on sequential application of LRT to study the origins of structural changes undergone by protein molecules. Similar approaches have been adopted in other work, whereby the perturbations on residues are introduced by modifying the effective force constants [21] or distances [22] between contacting pairs.

PRS relies on systematically applying forces at singly selected residues and recording the linear response of the whole protein. The response is quantified as both the magnitude of the displacements undergone by the residues, and their directionality. Closed form expressions that summarize the theoretical implications of the PRS technique in the limit of a large number of perturbations introduced at a given residue are provided. We note that we have previously studied the stability of proteins using a similar sequential perturbation-response approach, based on inserted displacements followed by energy minimization of the system [9],[23]. Therein we have also shown that the response of the system is within the linear regime for local distortions of atoms up to ca. 1.5 Å, despite the large local forces brought about [9].

Using PRS, we analyze the ferric binding protein A (FBP) as an example system, and describe alternative approaches that may have evolved in the structure to control function. The validity of the methodology is supported by molecular dynamics (MD) simulations. FBP is involved in the shuttling of Fe^+3^ from the mammalian host to the cytoplasm of pathogenic bacteria. To make iron unavailable to such pathogens, host organisms have iron transport systems such as the protein transferrin that tightly sequester the ion. Pathogens have developed strategies to circumvent this approach, one of them being the development of receptors for the iron transport proteins of the host. FBP resides in the periplasm, and receives iron from these receptors to eventually deliver it to the cytosol [24]. The protein is made up of two domains characteristic of periplasmic Fe^+3^ binding family as well as the host protein transferrin. These host/pathogen iron uptake proteins are thought to be distantly related through divergent evolution from an anion binding function.

Fe^+3^ is bound to FBP with remarkable affinity, with association constants on the order of 10^17^–10^22^ M^−1^ depending on the measurement conditions [25]. It was recently shown that a relatively high affinity of iron binding is required for the removal of iron from transferrin, and its transport across the periplasm [26]. Yet, this high affinity poses a Fe^+3^ transport dilemma, suggesting another necessary step for the release of the ion. It is of interest to understand how Fe^+3^ is eventually released from the binding site for subsequent use by the pathogen. One mode of action that has been suggested involves the control of the Fe^+3^ release kinetics by the exchange of synergistic anions forming relatively stable intermediates [25],[27],[28]. Another involves the direct action of chelators on the ion [25]. It has also been shown that mutants of FBP that are defective in binding the synergistic anion are still capable of donating iron, suggesting the possibility of still other alternative mechanisms for the process [29].

FBP is referred to as bacterial transferrin due to the similarities with transferrin in the structural folds, the highly conserved set of iron-coordinating residues, and their usage of a synergistic anion [30]. They do, however, differ in size, transferrin being made up of two-lobes having high sequence identity with each other (e.g. 45% in human transferrin). Each lobe itself is comparable to FBP in size, fold, and iron binding location. In transition from the open to the closed form, only one of the sub-domains in each lobe undergoes significant reorientation, similar to FBP [31]. Despite the resemblance, the iron binding/release kinetics in the two lobes differ. It has been implicated that there may be several approaches used for iron release in transferrins, including chelating agents and synergistic anions acting directly around the ferric binding site [32]–[35]. Additionally, it has been shown that chloride and other ion concentrations are effective on the kinetics, and it has been proposed that allosteric anion binding sites that trigger large conformational changes exist [10],[36],[37]. Based on the similarities between FBP and transferrin, it is of interest to find out if these routes also exist for FBP, and if they do, what the details of the mechanisms are. It is also of significance to determine possible binding locations on the surface as well as to understand the physical origin of such control.

In the current work, we study FBP in detail due to an extensive literature on the iron uptake mechanisms of this and evolutionarily related proteins; moreover, the molecular dynamics (MD) results of the apo structure have previously been analyzed by perturbing a singly selected residue with linear response theory [19]. We develop the PRS scheme such that, (i) we systematically apply LRT by scanning every residue on the protein so as to discriminate between residues that have major contributions to the biologically significant displacements, measured by x-ray experiments; (ii) we provide closed-form expressions for both the magnitude and the directionality of the response; (iii) we carry out further analysis of the response to uncover regions in the protein where coherent responses occur. Our findings are in agreement with a model where iron uptake by the protein is a favored process in the fluctuating environment, while iron release is specifically managed through several mechanisms including chelation and allosteric control. Furthermore, our findings suggest additional locations on the protein surface, far from the binding site, for allosteric control of iron release. The observations fit the “conformational selection” model whereby the existence of a weakly populated, higher energy conformation that is stabilized in the presence of the ligand is proposed [38],[39]; the hypothesis has recently been supported by NMR experiments by studying the protein structural ensemble of up to microseconds [40].

## Methods

The new tool introduced in this work for the analysis of remote control strategies utilized by proteins is based on applying forces at a given residue as a perturbation, and recording the displacements of all the residues as the response. Since the procedure is repeated sequentially for all the residues in the protein, we term the technique, perturbation-response scanning (PRS). Below, we first review the theory and then outline the details of the PRS technique. Finally, we describe the MD simulations.

### 

#### Linear response theory

**Figure 1:**
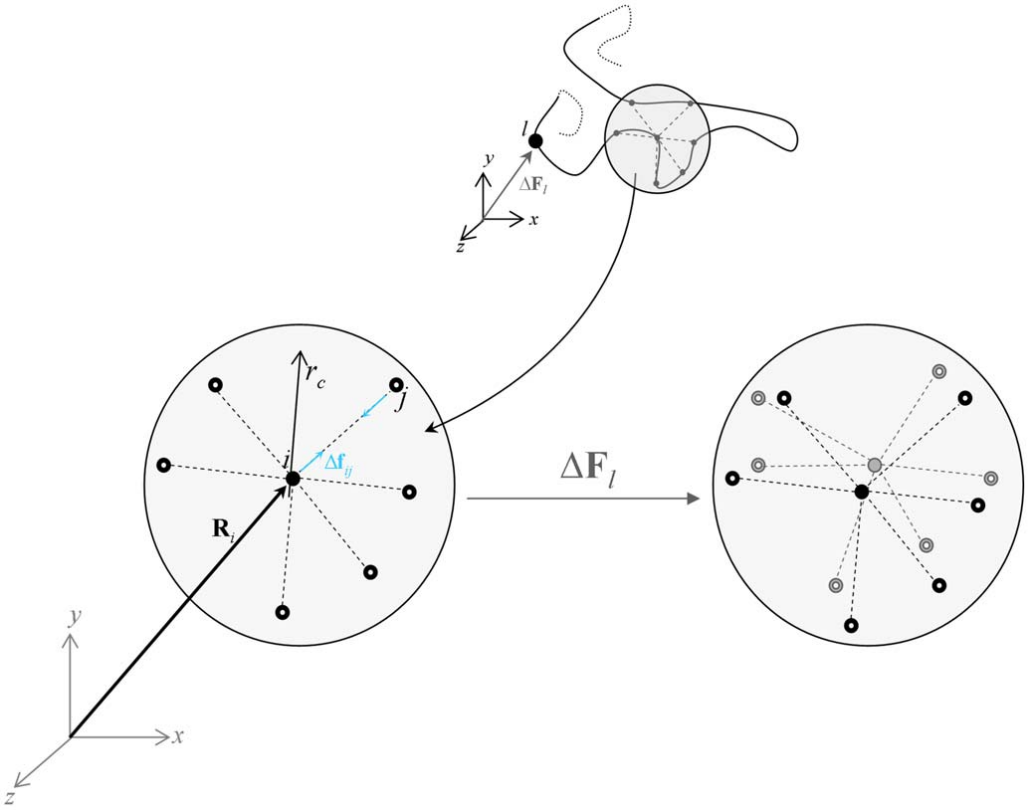
Free-body diagram of a residue. Excerpted from the protein chain (upper panel), scheme depicting the free body diagram of a C_α*i*_ atom coordinated by C_α*j*_'s within a cut-off radius *r_c_* (lower left). Δf*_ij_* denotes the interaction force between *i* and *j*. Under an external force applied on residue *l*, ΔF*_l_*, the residues are displaced in space (from the black to the gray nodes in the lower right). The contacting pairs are assumed not to change under this force.

Here we present a derivation of how a structure may be manipulated by external forces [41],[42]. We construct the protein as a residue network of N nodes that are centered on the *C*_α_ atoms. Any given pair of nodes are assumed to interact via a harmonic potential, if they are within a cut-off distance *r_c_* of each other (Figure 1).

In the notation used, **r** and **f** refer to the bond and internal force vectors along the edge connecting any two nodes, respectively. On the other hand, **R** and **F** are vectors on the nodes and are referred to as the position and external force vectors, respectively. There are *m* interactions pertaining to each residue (Figure 1, as an example, schematically illustrates the interactions for a residue that has six interactions, *i.e.*, *m* = 6), and a total of *M* interactions for the system of N residues. In the absence of an external force acting on the system, the equilibrium condition for each residue, *i*, necessitates that the summation of the internal, residue-residue interaction forces must be zero for each residue. Therefore,
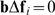
(1)where the 3×*m* coefficient matrix **b** consists of the direction cosines of each force representing the residue-residue interaction. The row indices of **b** are *x*, *y*, or z. Here Δ**f** *_i_* is an *m*×1 column matrix of forces aligned in the direction of the bond between the two interacting residues. For instance, in Figure 1, residue *i* has six contacts; and, thus, Δ**f** *_i_* is a 6×1 column matrix. Following the example outlined in Figure 1, equation 1 sums up the projection of these six forces on the *x*, *y*, and z-axes. This algebra gives rise to three independent equations involving six unknown interaction forces, which are the residual interaction forces of residue *i* with its contacting neighbors. One can write the equilibrium condition (equation 1) for each residue. This results in a total of N sets of equations, each of which involves the summation of forces in three respective directions. Consequently, generalizing equation 1 to the whole system of N nodes and a total of *M* interactions, one can write the following algebraic system of a total of 3N number of equations consisting of *M* number of unknown residue-residue interaction forces
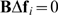
(2)with the 3N×*M* direction cosine matrix **B** and the *M*×1 column matrix of residue-residue interaction forces, Δ**f**. It is straightforward to generate the matrix **B** from the topology of the native structure (*i.e.*, the protein data bank (PDB) file[43]) with a specified *r_c_*. As an example, apo FBP has 309 residues and a total number of 1542 interactions when the cut-off distance of 8 Å is selected.

In the presence of an external force, Δ**F** (Figure 1), the equilibrium consideration for each residue dictates that the summation of the residue-residue interaction forces for each residue must be equal to the external, applied force on the same residue. Then, the equation 2 may be cast into the following form
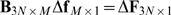
(3)Under the action of external forces, each residue experiences a displacement, Δ**R**, which is termed the positional displacement vector. Moreover, the bond distance between any two residues changes in the amount of Δ**r** in accord with the positional displacements of the two residues which participate in the bonding. Therefore, there must be compatibility between the total of 3N number of positional displacements and the changes that take place in the intra-residual distances, a total of *M* number of distortions. This compatibility is very similar to the form given in equation 3 [42]:
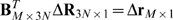
(4)Within the scope of an elastic network of residues that are connected to their neighbors with linear-elastic springs, the residual interaction forces, Δ**f**, are related to the changes in the contact distances, Δ**r**, through Hooke's law by
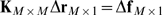
(5)where the coefficient matrix **K** is diagonal. Inasmuch as the native structures are stabilized predominantly by homogeneous tertiary contacts rather than specific interactions [44] we take the entries of **K** to be equivalent in this work. Note that we validate this assumption by comparing the residue cross-correlations obtained from the simplified Hookean potential in equation 5 with those from all atom MD simulations (see the subsection **Molecular Dynamics simulations** below, and **Results** **and** **Discussion** for details).

Thus, rearranging equations 3–5, one gets the forces necessary to induce a given point-by-point displacement of residues:
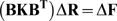
(6)On the other hand, one may choose to perturb a single or a set of residues, and follow the response of the residue network through,
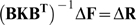
(7)where the Δ**F** vector will contain the components of the externally applied force vectors on the selected residues. The (**BKB^T^**) matrix is equivalent to the Hessian [41] and its inverse has six zero eigenvalues, corresponding to the global translational and rotational degrees of freedom of the system. The elements of the inverse of the Hessian, **G** = **H** ^−1^, may be used to predict the auto- and cross-correlations of residues. **G** may be viewed as an N×N matrix whose *ij*th element is the 3×3 matrix of correlations between the *x*-, *y*-, and *z*-components of the fluctuations Δ**R** *_i_* and Δ**R** *_j_* of residues *i* and *j*; *i.e.*,
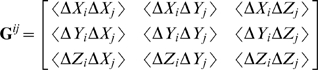
(8)The cross-correlations between residue pairs are obtained from the trace of its components:
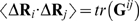
(9)Equation 9 has been shown to reproduce the cross-correlations obtained from MD simulations and molecular mechanics [9],[23]. In this work, we shall not be directly interested in the correlations, but rather shall use **G** as a kernel to predict the response of other residues to applied perturbations on selected ones as we discuss next.

#### Perturbation-response scanning

Our detailed PRS analysis is based on a systematic application of equation 7. We apply a force on the C_α_ atom of each residue by forming the Δ**F** vector in such a way that all the entries, except those corresponding to the residue being perturbed, are equal to zero. For a selected residue *i*, the force Δ**F** *^i^* is 
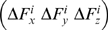
so that the external force vector is constructed as
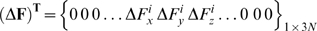
(10)The direction of the applied force vector deserves special attention. Here we choose the forcing direction randomly, attributing no bias due to the specific contact topology or the solvent exposed nature of the residue being perturbed. The forcing directions are uniformly distributed within a sphere enveloping the residue; therefore, the forcing may well be termed isotropic. It is definitely possible to favor specific directions leading to anisotropy in forcing, since there are no intrinsic constraints in the methodology dictating the opposite. A plausible forcing scenario for contact with a ligand, similar to one in [19] may also be conceived to determine the associated conformational changes. For specific applications, such as pulling experiments in which the application point and the direction of the forcing is identified (see, e.g. [45]), we can simulate the experimental loading conditions. If one does not explicitly know where such forces arrive at, however, the response of the different locations of the molecule under the action of a virtually applied isotropic force field is monitored by scanning the whole protein.

We then compute the resulting (Δ**R**) vector of the protein through equation 7, as we explain in detail in the following. Let the elements of **G** in equation 8 be *g_lm_* where *l* and *m* denote the indices for the second order partial differential of the total energy with respect to the directionality (*l*, *m* = *x_j_*, *y_j_* or z_j_; *j* is the residue index.) When a force is applied only at residue *i*, equation 7 in expanded form becomes:
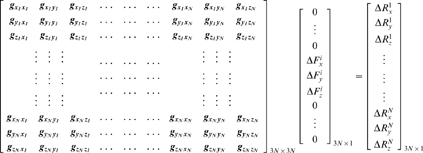
(11)The right-hand-side vector gives the displacement recorded in all the residues in response to a perturbation at a selected residue *i*,
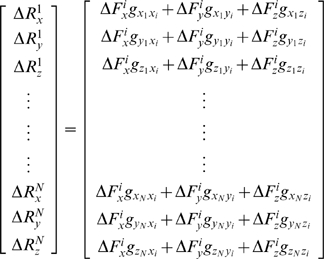
(12)Thus, a perturbation operates on the 3N×3 super-column of **G** that belongs to the residue being perturbed. The response on a specific residue *k* due to this perturbation on *i* is the vector Δ**R** *^ki^*

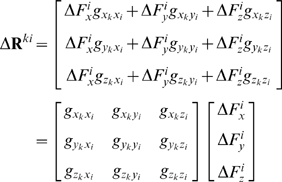
(13)We shall denote the 3×3 matrix of correlations between *i* and *k* by **G** *^ki^* (see also equation 8).

One may further obtain a theoretical limit on the displacements, as we shall show below. The elements and properties of **G** *^ki^* may be used to predict the average response of the system. First we apply a large collection, say *r*, of forces in random directions on residue *i* with mean zero and variance 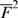.

The average displacement on residue *k* in the *x*-direction is
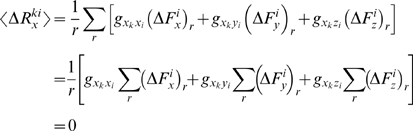
(14)since the application of forces in an uncorrelated fashion dictate 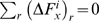 and similarly for the *y*- and z-directions. Here the average over any variable *a* is defined as 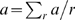. The average *magnitude* of the responses, on the other hand, is related to the applied forces and the elements of **G** through,
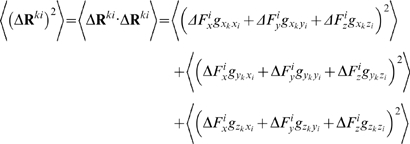
(15)In the expansion of the above expression, the average over any of the cross terms is zero, e.g.
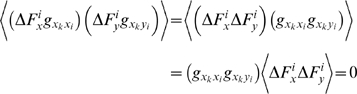
(16)Here, the elements of **G** factor out, as they are constants. Moreover, 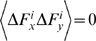 since the forces are applied in an uncorrelated manner. The self terms, on the other hand, survive to yield:
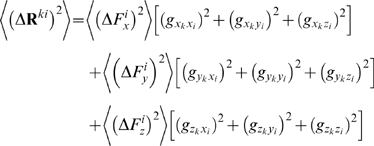
(17)with 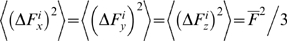 we have
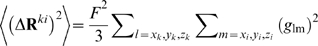
(18)Thus, the average expected displacement of a residue *k* due to the bombardment of another residue *i* may be calculated from the sum of the squares of the elements of the **G** *^ki^* matrix.

#### Correlations between predicted and experimental x-ray structures

**Figure 2:**
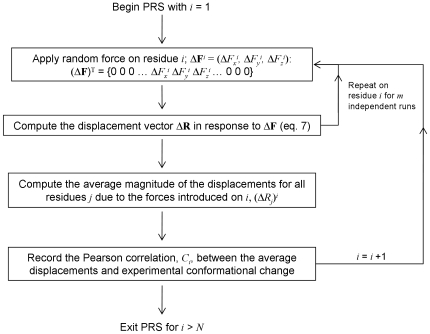
Algorithm A describing the overall PRS scheme.

Using PRS, we scan the protein by consecutively perturbing each residue, and record the associated displacements as a result of the linear response of the protein. We define 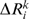 as the theoretical predictions of the displacement of each residue, *k*, as a response of the system to applied forces on residue *i* (equation 13). Similarly, Δ*S_k_* are the displacements between the apo and the holo forms obtained from the PDB structures (these are also referred to as the experimental conformational changes or experimental displacements throughout the text). Thus, 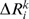 are compared with Δ*S_k_* and the goodness of the prediction is quantified as the Pearson correlation coefficient for each perturbed residue *i*:
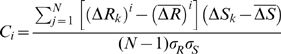
(19)where the overbar indicates the average. *σ_R_* and *σ_s_* are the respective standard deviations of calculated and experimental displacements. Gerstein and coworkers have demonstrated that when comparing two structures, the results from a selected subset of the residues may be more informative [46]. Thus, in calculating the correlations with the x-ray experiments, first a best-fit between the fixed domains of the predicted and the experimental structures are made. This is due to a generally used convention to interpret displacements in multi-domain proteins so as to accentuate the response of the moving domain to see the biological relevance of the observed changes. These calculations are repeated over at least five scans. The overall PRS scheme is summarized in Algorithm A, shown in Figure 2.

#### Analysis on the directionality of the response

If the collection of forces applied on a specific residue is independent and large in number, they will appear in a spherically symmetric set of directions. The responses, however, may be distributed along a line or in a plane so that the net response is still zero. Thus, although the perturbations are isotropic, the response may well be anisotropic. Deviations from such a spherically symmetric distribution of responses hint at the roles of certain residues in the remote control of the ligand, as will be shown in the **Results** **and** **Discussion** section.

For an analysis that probes the directionality of the recorded responses, we proceed as follows: We first concentrate on those residues for which the Pearson correlation between experimental and theoretical displacements is large. Amongst them, we further locate those residues, *i*, that are distant from the ligand binding site, *l*, (*i.e.*, the distance between them, *d_il_* » *r_c_*). For the selected residue, *i*, *k* forces are applied such that Σ*_k_* Δ**F** *_k_^i^* = 0; *k* is large and ensures that a spherically symmetric region around *i* is covered. The sum of the responses on each residue *j* is zero, Σ*_k_*Δ**R** *_k_^j^* = 0. The results are visualized as vector plots on the protein structure (as will be exemplified in the subsection **Local and remote modulation of Fe ion dissociation**).

One may further analyze the eigenvalue structure of the **G** *^ki^* submatrix to have an understanding on the nature of the response by decomposing it using the transformation
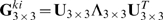
(20)The three orthogonal vectors defined by **U**, **u** *_j_*, give the three principal axes of the line of action of residue *k* in response to perturbations in *i*. The size of the associated elements, *λ_j_*, provide the contribution, 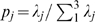, of each vector to the overall response to the perturbation Δ**F** *^i^* (equation 13). Thus, if there is one dominant eigenvalue in **G** *^ki^*, i.e., 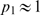, then no matter what the values of the elements of Δ**F** *^i^*, they will be projected on the associated eigenvector, **u**_1_. Therefore, the collection of responses Δ**R** *^ki^* to a number of perturbations will all be collected in a line along **u**_1_. Similarly, if two eigenvalues dominate, *i.e.* 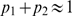, then the collection of responses will occur in the plane defined by **u**_1_ and **u**_2_.

**Figure 3:**
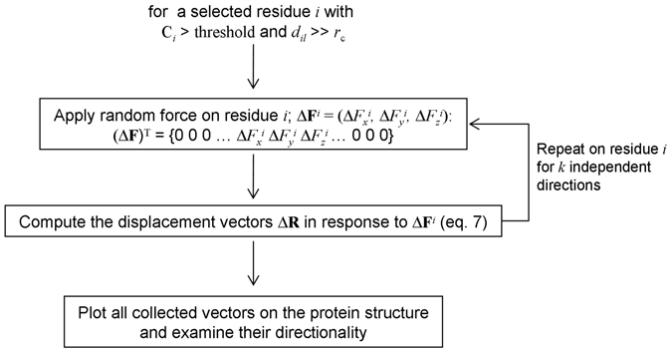
Algorithm B describing the directionality analysis.

The degree of collectivity of the response of a group of neighboring residues to a perturbation on *i* may also be measured using equation 20. If the response of the neighbors possesses collectivity, then various symmetries in their action may be expected. For example, if the residues collectively move in a line to open a cap, not only each is expected to have a single dominant eigenvalue, but also the eigenvectors belonging to these dominant eigenvalues are to be parallel; *i.e.*,
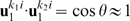
(21)where *θ* is the angle between the two eigenvectors, and *k*_1_ and *k*_2_ are the two residues whose responses are being compared. The directionality analysis is summarized in Algorithm B, shown in Figure 3.

#### Systems studied and residue network construction

We analyze FBP in detail, using both PRS and MD. In addition, the PRS methodology is applied to several other cases to demonstrate how the approach may be used. These are included in Text S1.

**Figure 4:**
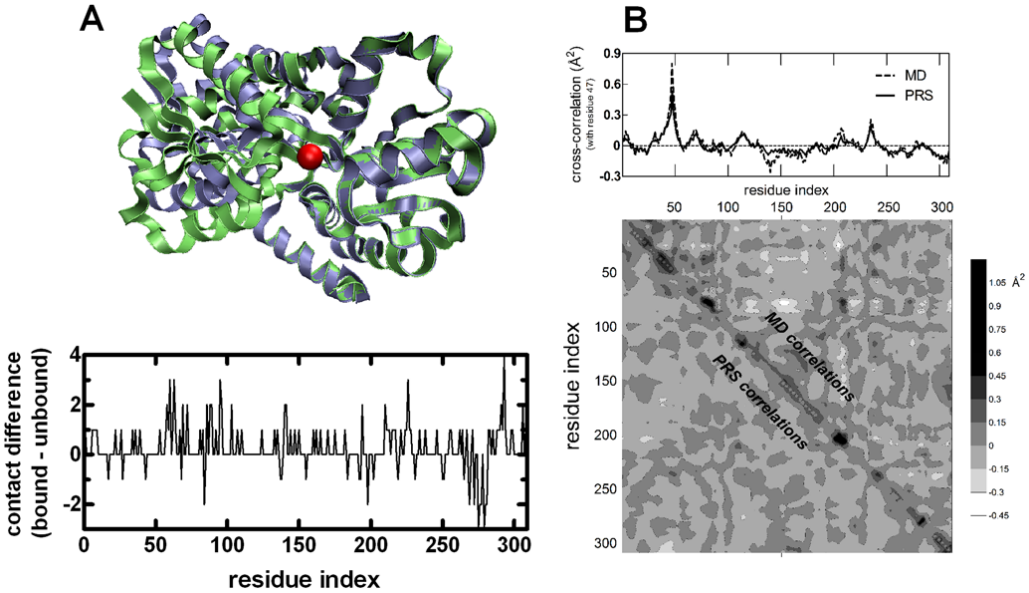
Analysis of ferric-binding protein. **A** Upper panel displays the haemophilus influenzae ferric-binding protein in apo (purple; PDB code: 1D9V) and holo forms (green; PDB code: 1MRP). The two structures are superimposed on the fixed domain (residues 83–87, 102–225, 277–307). The Fe^3+^ ion is shown as a red sphere. Residues 9, 57, 175 and 193 are within 7 Å of the Fe atom. In addition, residues 8, 139–141, 176, 195 and 196 are in its 7–8 Å range. The lower panel shows the difference between the number of contacts of the ferric bound and unbound forms of FBP. **B** Contour map comparing the residue cross-correlations obtained from the detailed MD simulations of 10 ns duration (upper-right part of the map) and the coarse-grained model (lower-left part of the map) for holo-FBP. White and light gray are negative correlations, dark gray and black are positive correlations. Also displayed at the top of the figure are the detailed cross-correlations of residue 47 from the coarsened protein model (solid lines) and MD simulations (dashed lines). The value of zero is marked for each case to better discriminate positively and negatively correlated regions. The Pearson correlations between the data are 0.74 for the whole map and 0.94 for the selected residue, respectively.

The apo and holo forms of FBP have PDB codes 1D9V and 1MRP, respectively; in the latter case the Fe ion is treated as an additional node of the network. The protein has two domains, and upon binding one moves relative to the other as shown in figure 4a. Unless otherwise specified, the final structures are superimposed on the fixed domain of the initial structure (residues 83–87, 102–225, 277–307), before the displacements are computed by equation 7 or 11. Thus, we ensure that the response that is reflected on the collective motion of the fixed domain is removed. Note, however, that this is done only to achieve a quantitative comparison with the all-atom study in reference 19 and the overall conclusions of the study are not affected by this choice. When no superimposition is applied, the values of the correlations, *C_i_* (equation 19) are lower in value, but their relative ordering does not change; for example, the largest correlations in the apo form reduce from 0.98 to 0.90 and those in the apo form reduce from 0.89 to 0.80.

As a cut-off distance, we seek the smallest value for which the experimental B-factors are well-reproduced (equation 9 with *i* = *j*) and the results are the same over a range of values. We find that a cut-off distance of *r_c_* = 8.0 Å on the C_α_ atoms of the protein (equation 1) is suitable. We have also verified that the main conclusions of the study are not affected for a range of values between 7.0–8.5 Å; the system exhibits six zero eigenvalues at all these cut-off distances. At *r_c_* = 8.0 Å, there are 1542 and 1587 interactions for the apo and holo initial conformations, respectively.

#### Molecular dynamics simulations

To provide a basis for comparison with results from an all-atom approach, we have performed MD simulations on both the apo and the holo forms of FBP in water. The systems are prepared using the VMD 1.8.6 program with solvate plug-in version 1.2.[47] The NAMD package is used to model the dynamics of the protein – water systems.[48] The apo form is neutral with 309 amino acids, and an exogenous phosphate ion. The holo form additionally has the Fe^+3^ ion. Three chloride ions are added to achieve charge neutrality in the latter system. The protein is soaked in a solvent box such that there is at least a 5 Å layer of solvent in each direction from any atom of the protein to the edge of the box. The simulated protein-water complexes have 5156 and 5368 water molecules, respectively for the apo and holo forms. The CharmM27 force field parameters are used for protein and water molecules [49].

The binding site parameters are as follows: The exogenous phosphate is modeled in the H_2_PO_4_^−^ state using the parameters reported in detail in the literature [50]. For the Fe^+3^ ion, an effective van der Waals interaction term in addition to electrostatics is included in the spirit done for other ions in the literature [51]. Since the parameters for Fe^+3^ do not appear in literature, we have self-consistently parameterized them so that the six liganded coordination within 2.0±0.2 Å average distance of the ion [52] is maintained after energy minimization and 200 ps long MD simulations. The optimal values of the Lennard-Jones parameters were found to be −0.1 kcal/mol for well-depth and 2.6 Å for the separation at the minimum.

Long range electrostatic interactions were calculated using particle mesh Ewald (PME) method [53]. The cutoff distance for non-bonded van der Waals interactions was set to 12 Å with a switching function cutoff of 10 Å. Rattle algorithm was used to fix the bond lengths to their average values. During the simulations, periodic boundary conditions were used and the equations of motion were integrated using the Verlet algorithm with a step size of 2 fs [54]. Temperature control was carried out by Langevin dynamics with a dampening coefficient of 5/ps and pressure control was attained by a Langevin piston. Volumetric fluctuations were preset to be isotropic in the NPT runs.

Both systems were first subjected to energy minimization with the conjugate gradients algorithm until the gradient tolerance was less than 10^−2^ kcal/mol/Å. 500 ps MD runs in the NVT ensemble at 310 K were carried out on the resulting systems. The final structures were then run in the NPT ensemble at 1 atm and 310 K until volumetric fluctuations were stable to maintain the desired average pressure. This process required 500 ps long MD runs at the end of which the average volume is maintained at 196900±700 and 203300±600 Å^3^ in the apo and holo structure runs, respectively. Finally, the runs in the NPT ensemble were extended to a total of 10 ns. The coordinate sets were saved at 2 ps intervals for subsequent analysis.

The RMSD of the trajectories were calculated (Figure S1). For the holo form stabilized by the Fe ion, equilibration is reached within 500 ps, with the value 1.4±0.1 Å averaged over the remaining 9.5 ns trajectory. On the other hand, the RMSD of the apo form displays larger fluctuations, and the point of equilibration is harder to judge for this form. The RMSD is 1.6±0.3 Å averaged over the last 9.5 ns trajectory, and 1.8±0.2 averaged over the last 5.0 ns. The results presented in this manuscript are those obtained from the last 5.0 ns of the simulations for both forms of FBP. Note that, we have repeated the analyses for the last 9.5 ns of the trajectories, without any significant difference in the results.

The correlations between residue pairs derived from the MD trajectories are of particular interest. The snapshots recorded during the MD simulations are organized in the fluctuation trajectory matrix of order 3N×T, [23]

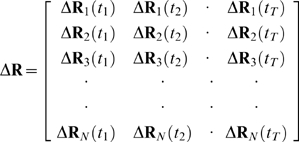
(22)The 3N×3N correlation matrix is then calculated by 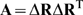, where T is the transpose. The cross-correlation between residue pairs are then computed through equations 8 and 9, where **A** substitutes for **G**.

Note that a each nanosecond of an MD simulation takes ca. 9.8 hours on a server with 2 GB memory and eight CPUs each with 2.4 GHz quadcore architecture. Five complete scans of the protein with the PRS method uses three minutes on a single CPU of the same server.

## Results

Based on linear response theory, we apply perturbations at selected points along the chain to diagnose the response of FBP. The protein is known to have a Fe^3+^ binding location, and the structure of the holo form is also known. The overall RMSD between the two forms is 2.48 Å. FBP has two domains, termed as the fixed and moving domains, respectively (in figure 4a the two structures are superimposed on the fixed domain residues: purple apo-, green holo-FBP). The difference in the contact numbers of the two forms is shown in the bottom panel of figure 4a. The main motion in the protein is hinge, with the moving domain residues closing up on the Fe binding location and the total number of contacts in the protein increasing from 1542 to 1576. The Fe ion brings in only 11 of the new contacts. For 111 residues, the contact number remains unchanged; others lose/gain up to three contacts, with the exception of residue 293 having four additional contacts (Figure 4a). The conformational change is mainly governed by a hinge motion around the Fe-binding location; yet, the two structures have distinct features. It is therefore of interest to see the extent to which the two conformations are inter-convertible, so as to describe the mechanisms governing the high affinity iron binding and its selective release.

### 

#### Validation of the coarse grained model

Since the methodology employs the Hessian obtained from a coarse-grained potential in equation 7, we first determine the extent to which this potential represents the actual interactions between residue pairs. We compare the correlations obtained from the network model with those from the MD simulations for both the apo and the holo forms (by applying equations 8 and 9 for the former, and equations 22, 8, and 9 for the latter). We find that the coarsened protein model reproduces the residue-wise MD correlations; the Pearson correlations between the data are 0.76 and 0.74, respectively for the apo and holo forms. In Figure 4b we display a comparative map of the correlations obtained by the two approaches for holo FBP. A cross-section through a sample residue, Asp47 that resides on a flexible loop on the surface of the moving domain, is also displayed at the top of the figure. The two approaches agree well, including the regions of negative correlations, which lie below the solid and dashed lines for the respective cases of coarse-grained and MD results, and corroborate the results in reference 19. Note that therein equation 7 was computed for perturbations introduced to Glu57 only. In the next subsection, we elaborate on the results obtained for this residue, which directly contacts the ion in the holo structure.

**Figure 5:**
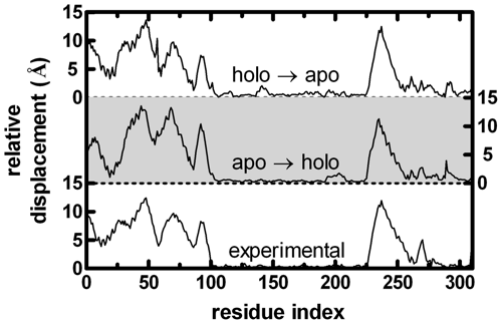
Relative displacements of residues between the apo and holo forms (x-ray), and typical responses to a given force perturbation on residue 57 in the apo and holo forms. The latter two curves are nudged for ease of comparison; their baselines are shown by dashed lines. Since the calculated displacement is proportional to the imposed force in LRT and therefore may be rescaled by a proportionality constant, the magnitude of the force is adjusted so as to make the average displacement the same as that of the x-ray experiment.

The relative magnitudes of the residue-by-residue displacement vectors between the experimental apo – holo structures after superimposing their fixed domains is shown in the bottom curve of Figure 5. It was previously shown that applying forces on residue 57 near the ferric binding site yields the expected atomic displacements.[19] Therein, the correlation coefficient between the theoretical and experimental relative displacements, computed from the displacement vectors between the holo and apo forms, was reported to be 0.95. Example cases of the computed displacements using equation 7 are shown in the middle and upper curves of Figure 5. Here, a single perturbation on residue 57 is placed in a randomly chosen direction on the apo and holo structures, respectively, and the fixed domains of the resulting structures are superimposed. The correlation coefficient, *C*_57_ (equation 19), between these curves and the experimental curve is 0.95 and 0.92, respectively for these example cases. The close agreement of the residue-by-residue displacements between the current methodology and the all-atom approach in reference 19 justifies the assumptions that (i) the Hessian obtained from the elastic network adequately describes the system; (ii) it suffices to take the contacts to be homogeneous (*i.e.*, the **K** matrix in equation 7 is identity). We next investigate whether residue 57 is unique in reproducing the conformational response of the protein by performing PRS.

#### Non-reciprocal response to localized perturbations throughout the protein uncovers landscape properties

**Figure 6:**
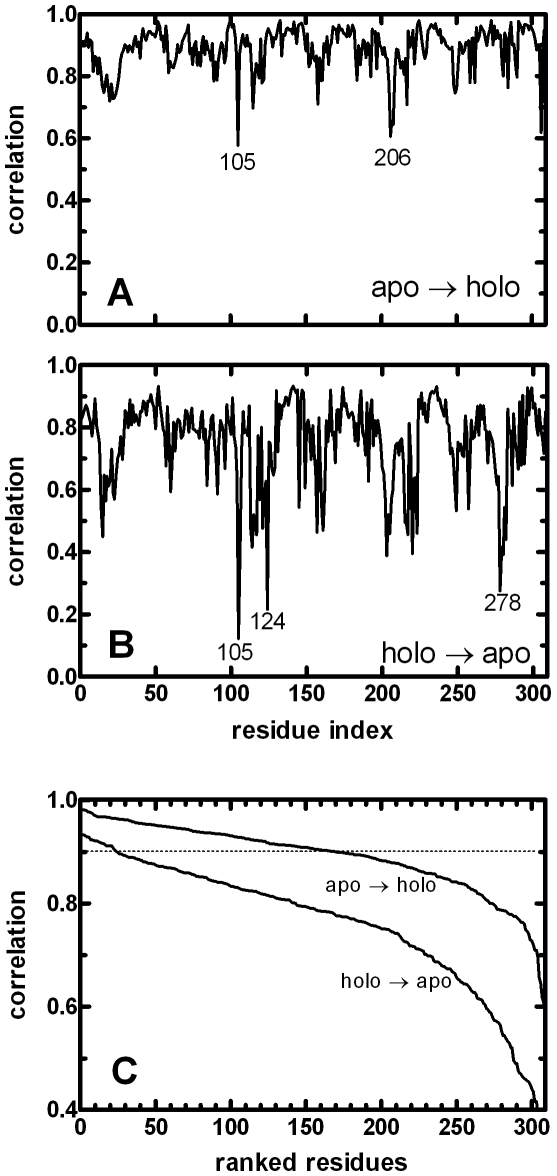
Displacement vectors between the perturbed and initial structures of ferric-binding protein. The displacements are compared with those of the crystal structures, for **A** the apo, and **B** the holo form as the initial structure. In **C**, data in **A** and **B** are sorted from larges to smallest. Residues 47, 52, 130, 139–144, 147, 148, 166, 174, 186, 226, 232–236, 293, 296, 298, 299 give the highest and 105, 124, 278 the lowest correlations in the holo structure. The standard error on the mean increases with decreasing correlations, as determined from the averages of five perturbations in randomly selected directions. For correlations greater than 0.90, it is less than 0.05 for the apo, and 0.02 for the holo form; the largest errors on the mean are 0.2 for the least correlated values in both cases.

We now perform the residue-by-residue scan on the protein. Starting from each of the initial conformations, (i) apo form and (ii) holo form with the Fe ion as an additional node in the network, we compute Δ**R** for each residue and record the response obtained as outlined in the subsection **Correlations between predicted and experimental structures**. In figure 6 we display the resulting correlation coefficients *C_i_* between the calculated and the experimental data for every residue (equation 19). Note that each point on these figures is the result of the comparison of the displacements of the 309 residues in response to a perturbation applied at the selected residue, *i*, such as that obtained from the correlation between the middle and bottom curves in figure 5. We find that there is a plethora of residues whose perturbation leads to the holo structure when applied on the apo structure, whereby *all* applied forces lead to displacements well correlated with those from x-ray structures, with the worst perturbation having a correlation of 0.6±0.1 (figure 6a). In fact, on average, perturbing 169 of the 309 residues (55%) of this protein lead to displacements that are correlated with a coefficient of 0.90 or better with experiments. When we perform PRS on the holo form as the input structure, only 24 out of 309 residues (8%) that perform better than 0.90 correlation remain (figure 6b). To display the reduction in the number of residues that lead to favorable conformational change upon perturbation, the correlations in figure 6a and b are ranked according to their size, and are plotted in figure 6c.

In perturbing the residues of the apo FBP, the residues that give the worst correlations are 105 and 205–207, all in the fixed domain of the protein. Residue 105 is in the core of the β sheet structure located in this domain and 205–207 are at the turn adjoining a helix to a β strand. Additional residues with the largest deviations between the experiments and predicted values of the structural differences are due to perturbations in the fixed domain. Finally, residues 23 and 249, located in the core of the moving domain also lead to poorer predictions. These relatively low-correlation responses (*C_i_* = 0.58–0.75) are not due to high coordination numbers of the involved residues which span a wide range of 4–13 contacts, implying a more intricate set of interactions leading to these results.

In perturbing the residues of the holo FBP, the number of residues that leads to a highly correlated Δ**R** profile with the crystal structure is reduced. The residues whose perturbation leads to displacements with the largest *C_i_* [*i.e.*, those that lie above the dotted line] and the lowest *C_i_* are listed in the caption to Figure 6. All of the latter are located in the fixed domain. The Fe ion is known to bind FBP with very high affinity, assisted by the coordinating phosphate ion. Those with high *C_i_* values are not distributed throughout the structure, but are clustered in two α-helices in the fixed domain, and on loops in the moving domain. Thus, once the Fe ion is bound, the structure becomes stabilized such that it can be moved towards the apo form only through perturbing specific residues. Direct force application at the Fe ion itself leads to an average correlation coefficient of 0.88.

The high *C_i_* values resulting from the perturbation of many residues on the apo structure merely suggest that the motions are *directed* towards the holo form. Whether it will actually inter-convert to that structure depends on the energy barrier between the two forms. It may further be that the holo form may only be stabilized in the presence of its ligand. The existence of a weakly populated, higher energy conformation that is stabilized in the presence of the ligand, so that the equilibrium is shifted towards this minor species has been experimentally observed,[40] supporting the “conformational selection” hypothesis.[38] This viewpoint of “shifts in the energy landscapes” was put championed a decade ago by Nussinov and coworkers [39],[55]. It is highly probable that following an applied perturbation on any given residue, the structure will originally tend towards the holo conformation, but will return to the average apo conformation before reaching the holo state due to the incessant bombardment from the surroundings. Yet, as depicted in figure 6a, most of the perturbations applied at a variety of residues distributed throughout the protein lead to very high correlations with the displacements obtained from x-ray structures. This implies that the conformational changes attempted by the apo form of the protein in its dynamical environment are narrowly distributed, and tuned towards the holo form. These results further suggest that the apo and holo forms are probably closely located along the free energy landscape. In the next subsection, we rely on the directionality information provided by the PRS to uncover the mechanisms that dictate protein function.

#### Local and remote modulation of Fe ion dissociation

To provide further understanding of how the protein operates structurally, we turn to the few residues that give high correlations in the presence of the ligand. These include residues that are either in the fixed domain that support the ferric binding region or those that are located in the moving domain loops. Thus, it is possible to manipulate the bound form of the protein towards the unbound form by either directly perturbing the Fe-binding residues, or by controlling the distant flexible loops. If there is a directionality preference in the response of residues, they should additionally be revealed by the current analysis. Such a preference may be imposed by the excluded volume, or may be the result of coupling between the motions of subsets of nodes.

In the previous subsection we have shown that in holo FBP, singly placed forces on the residues listed under the caption to figure 6 reproduce the displacement profile from x-ray structures with very high precision. The relative magnitudes of the displacements are correctly captured by this procedure. On the other hand, the recorded response also has a directionality that is concealed in these correlations. One measure of directionality is the overlap, defined as the cosine of the angle between the calculated and measured direction of motion. However, this definition eliminates information on the magnitude of the motion, and even if the applied methodology captures the essential motions of key residues, many others that have no preferred direction of motion blur the statistics. Therefore, the results from a selected subset of the residues may be more informative, as demonstrated by Gerstein and coworkers [46]. In fact, we find that overlap distributions over all the residues are close to those of a random distribution of angles (results not shown). We therefore perform the following alternative analysis (see Algorithm B in Figure 3): For a selected residue, for instance, from those that have the highest correlations (*C_i_*>0.9), we introduce a large collection of perturbations in directions that are spherically symmetric around it, so that their vectorial sum is zero. For each perturbation, we monitor the resulting response in the residues directly contacting the Fe^+3^. The directional response is also analyzed analytically by applying equations 20 and 21.

**Figure 7:**
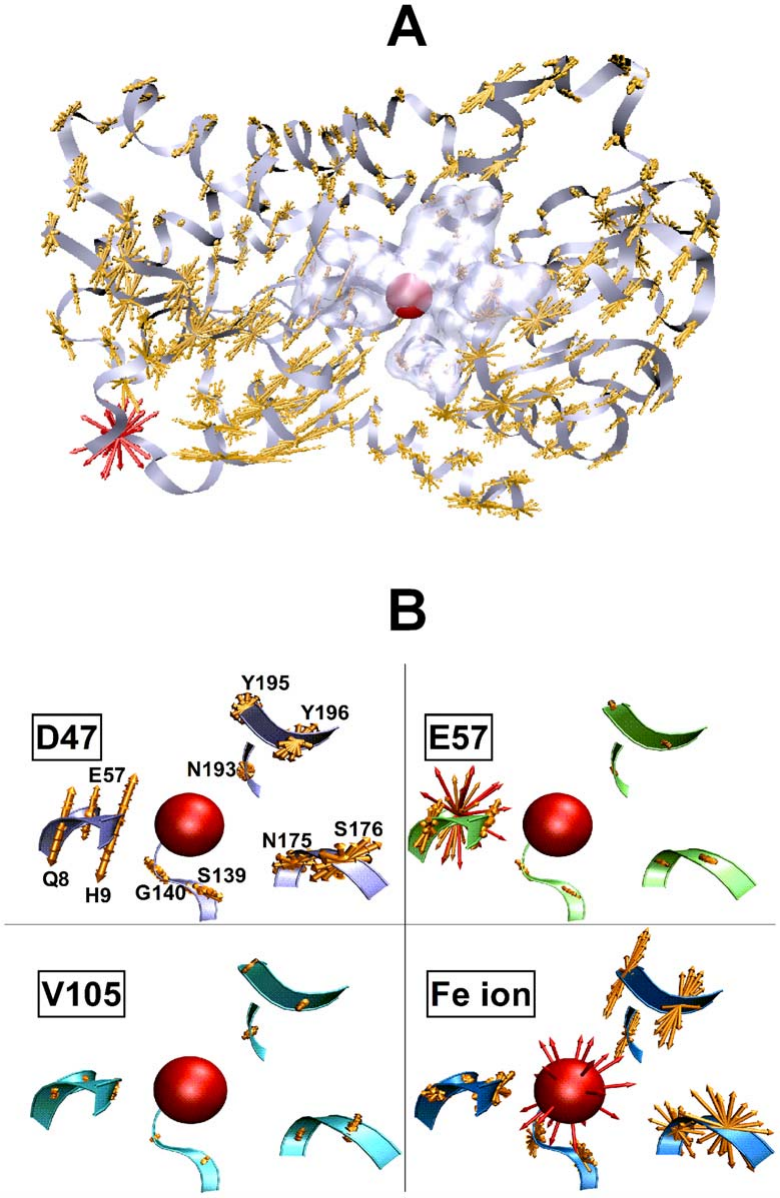
Remote modulation of Fe ion dissociation. **A** The response of the protein to forces exerted on residue *j* = 47 in different directions (forces are shown with the red arrows.) The collection of all the displacements is shown in orange; responses on the three neighbors along the chain in either direction (44≤*j*≤50) are not shown for clarity. The volume taken up by the first neighbors of the Fe^+3^ is shaded. **B** The region of the first neighbors of the Fe^+3^ is magnified to display the response to perturbations applied at selected residues. All vector lengths are relative to the magnitude of the same size of unit response. **Upper left** figure magnifies the results in **A**. The Fe^+3^ ion contacting residues in the fixed domain (located on the right hand side) respond incoherently, moving mainly within a plane. Residues Q8, H9 and E57, that are in the moving domain of the protein respond coherently, moving parallel to each other, tending to open the cap for Fe ion to exit. Perturbations on regions in the binding domain (exemplified by **E57** and **Fe ion**) also induce large changes in this region, the former in the moving domain, the latter in both domains, but coherence in cap residues no longer exists. Distant, non-controlling residues (e.g. **V105**) cannot induce large enough motions in this region, although coherence of the cap residues may exist (see Table 1). Figures are drawn with the VMD software [47].

The residues for which *C_i_*>0.9 and are far from the Fe ion (*d_i_*_-Fe_ » *r_c_*) are D47, D52, both at the tips of distant loops as well as the loop spanning 232–236. The results for the perturbations on D47 (*C_i_* = 0.91, *d*_47-Fe_ = 28.5 Å) are shown in figure 7a, where the applied forces are in red and the responses are in orange. We observe that many of the residues that have high displacements are found to move in a plane, due to constraints imposed by chain connectivity. Others, usually those with small magnitudes, move in a spherical region, whereas a few show movements along a line; response sets intermediate of a line and a plane are also observed. We further focus on the responses of the 11 residues whose C_α_ atoms are within 8 Å of the ion; three are located in the moving domain (residues 8, 9 and 57), and the remaining are in the fixed domain. The volume they take up in the protein is shaded in figure 7a. This region is magnified in figure 7b, where the recorded responses to selected perturbations are shown. The upper left panel of figure 7b focuses on the data in figure 7a to show that the moving domain residues contacting the Fe ion operate in a coherent fashion at the tip of the cap that opens the exit of the Fe ion. Here, we define coherence as the tendency of residues to move along parallel lines. The local eigenvector analysis described in the Methods section (equations 20 and 21) confirms that upon perturbing these highly correlating residues, the inverse of the Hessian operates on a single dominant eigenvalue (*p*_1_>0.8) whose associated eigenvectors are parallel to each other (cos *θ*>0.95). The coherence is also obtained for forces applied on residues 52 and 232–236 (*C_i_*>0.9 and *d_i_*_-Fe_>21 Å). We also perform the same analysis to the correlation matrix obtained from MD simulations (equations 22, 8 and 9). The results are summarized in Table 1 for perturbations on residues 47 and 52 where both PRS and MD predict coherent response with a single dominant eigenvalue and parallel eigenvectors.

**Table 1:** Response of cap residues 8, 9, and 57 to perturbations on selected residues.

	fractional contribution of the highest eigenvalue *p*_1_ of the response matrix G*^ki^* (*k*: 8/9/57)†		angle *θ* between average response vectors of residue pairs 8–9/8–57‡	
perturbed residue, *i*	PRS	MD	PRS	MD
D47	0.95/0.92/0.81	0.78/0.83/0.60	9/16	5/28
D52	0.88/0.90/0.81	0.91/0.95/0.79	9/14	4/18
E57	0.54/0.56/0.50	0.77/0.86/0.57	36/38	4/16
Fe ion	0.56/0.67/0.54	0.74/0.81/0.59	25/36	5/28
V105	0.73/0.77/0.62	0.89/0.88/0.92	7/11	6/19

**†:** As computed from equation 20.

**‡:** Computed through equation 21; relative response of cap residues in the moving domain, *k*, to perturbations in residue *i* is coherent if *θ* is small and *p*_1_ is large.

On the other hand, directly perturbing the Fe ion, as well as other local residues for which *C_i_*>0.9 and *d_i_*_-Fe_≈*r_c_*, destroys this coherence of the cap residues; *i.e.*, they move in a much wider range of directions as exemplified by perturbing Fe ion and residue 57 in the right hand side panels of figure 7b. These do not have dominant eigenvalues in the cap residues (Table 1) and the angles between the eigenvectors that belong to the largest eigenvalue of pairs of residues 8, 9 and 57 are larger than 30°, making the response in this region far from coherent. Analyses of MD results predict similar coherence in the correlations amongst the binding region residues compared to those between this region and the distant controlling sites.

In the lower left panel of Figure 7b, we display the response of the binding region residues to a collection of perturbations on residue 105, which gives the lowest correlations in holo FBP (recall Figure 6c). We find that the magnitude of the induced response is insignificant, although the cap residues act more coherently compared to local perturbations, as shown in Table 1; MD correlations also corroborate this tendency.

Thus, by jointly focusing on the specific distantly located residues which (i) invoke a large amount of correlations in the whole protein, and (ii) induce local cooperativity in the binding domain, one may be able to uncover sites that remotely control function in the protein. Interestingly, the same analysis carried out on the N-lobe of human transferrin implies K278 in that protein as an allosteric controller (see Text S1, section E). This residue resides in a bulging loop, and does not have a structurally aligned counterpart on bacterial FBP. These observations open the way to the design of agents that specifically target the pathogen, while the functionality of the host is not altered.

## Discussion

In this study we introduce the PRS methodology based on systematically perturbing all residues of a protein, and classifying both the magnitude and the directionality of the recorded linear response. The approach is unique in that the cross-correlations between residues are processed as input for further predictions on the system behavior, unlike other methods where they are the final results. Closed-form expressions for the magnitude and the directionality of the response are also provided. The protein FBP is studied in detail, and results using PRS for additional cases are presented in Text S1. All computer programs used in the analyses are available upon request. The findings on FBP are also supported by MD simulations.

For the particular case of FBP, our analysis suggest the existence of two alternative mechanism of Fe ion release in FBP: (i) Local control of the ion by synergistic anions and chelators acting in the binding groove, and (ii) remote control by ions acting on distant charged residues located in solvent exposed loops (*e.g.*, D47, D52, K234) due to their observed ability to mechanically control the cap over the ligand binding region. For FBP, the former type of control has been evidenced by a plethora of experiments where the exchange of synergistic anions forming relatively stable intermediates or the direct action of chelators on the ion have been observed [25],[27],[28]. There are no mutational studies on FBP directly implicating the distant residues mentioned in latter scenario. However, it was recently shown that *H. influenza* strains expressing mutant proteins that are defective in binding the phosphate anion are capable of donating iron, calling for mechanisms of iron transport that do not involve a synergistic anion.[29] Furthermore, the kinetic effect of chloride and perchlorate (which does not coordinate to Fe^+3^) has called for anion binding sites on the surface of FBP, similar to those found in transferrin [27]. Allosteric anion binding sites that trigger large conformational changes located at the surface [10],[36],[37] have been determined in the structurally and functionally similar protein transferrin. In particular, R124 located in the N-lobe of the latter has been found to control iron release rate by anchoring synergistic anions [32]. Structural alignment [56] of transferrin with FBP shows that this position is equivalent to F142 located in the helix supporting the Fe binding region; the latter is amongst the residues that result in the highest correlations with the experimental data upon perturbation. Similarly, K206 which provides anion binding sites in human transferrin N-lobe holds an equivalent position to that of E193 in FBP, the latter also showing high displacement correlation following its perturbation (figure 6).

The results resolve the so-called Fe^+3^ transport dilemma: The protein is ready to uptake the ion in the apo structure (figure 6a), but it is necessary to perturb highly specific locations along the chain for its release in holo FBP (figures 6b and 7). This mode of action provides a mechanism for recent NMR observations of ligand binding whereby the energy landscape of the free protein is made-up of a set of coupled low-free energy states [5],[40],[57],[58]. Therein, ligand binding is considered as the source for shifts in the landscapes [38],[39]. PRS confirms this view for the particular case of FBP, and further provides the mechanisms on how the ligand binding region may be manipulated, as outlined by scenarios (i) and (ii).

In summary, the PRS methodology introduces an efficient approach to determine regions in the protein that mechanically moderate binding region motions. It may therefore be used to determine candidate sites for mutational studies. In a forthcoming study, the biological implications from the results of PRS will be presented on set of twenty proteins that display various types of motions such as shear, hinge, allosteric, partial refolding as well as more complex protein motions, as classified in the Database of Macromolecular Motions [59].

Although studies such as the current one help estimate allosteric sites, they do not provide information on pathways along which the remote communication takes place. As future work, it is of interest to locate these paths. Robust techniques to predict them using evolutionary information [60] or the specificity of the interactions within the residue networks [61] have been developed. Cross-correlation information from MD simulations have been used with residue network properties [62] to generate information on remote communication pathways [60],[63],[64]. Complemented with point mutation studies, such analyses will not only aid the protein design process, but will also uncover the physics of remote communication between residues.

## Supporting Information

Figure S1 The RMSD values of the 10 ns long MD trajectories. The holo form is stabilized by the ligand, while the apo form displays larger fluctuations.
(0.10 MB DOC)

Text S1 Sample applications of the PRS method.
(1.45 MB DOC)
