# Supplementary figures and images for: Perturbation-Response Scanning Reveals Ligand Entry-Exit Mechanisms of Ferric Binding Protein
Source: PLoS Comput Biol. 2009 Oct 23;5(10):e1000544. doi: 10.1371/journal.pcbi.1000544 (PMC2758672; doi:10.1371/journal.pcbi.1000544)

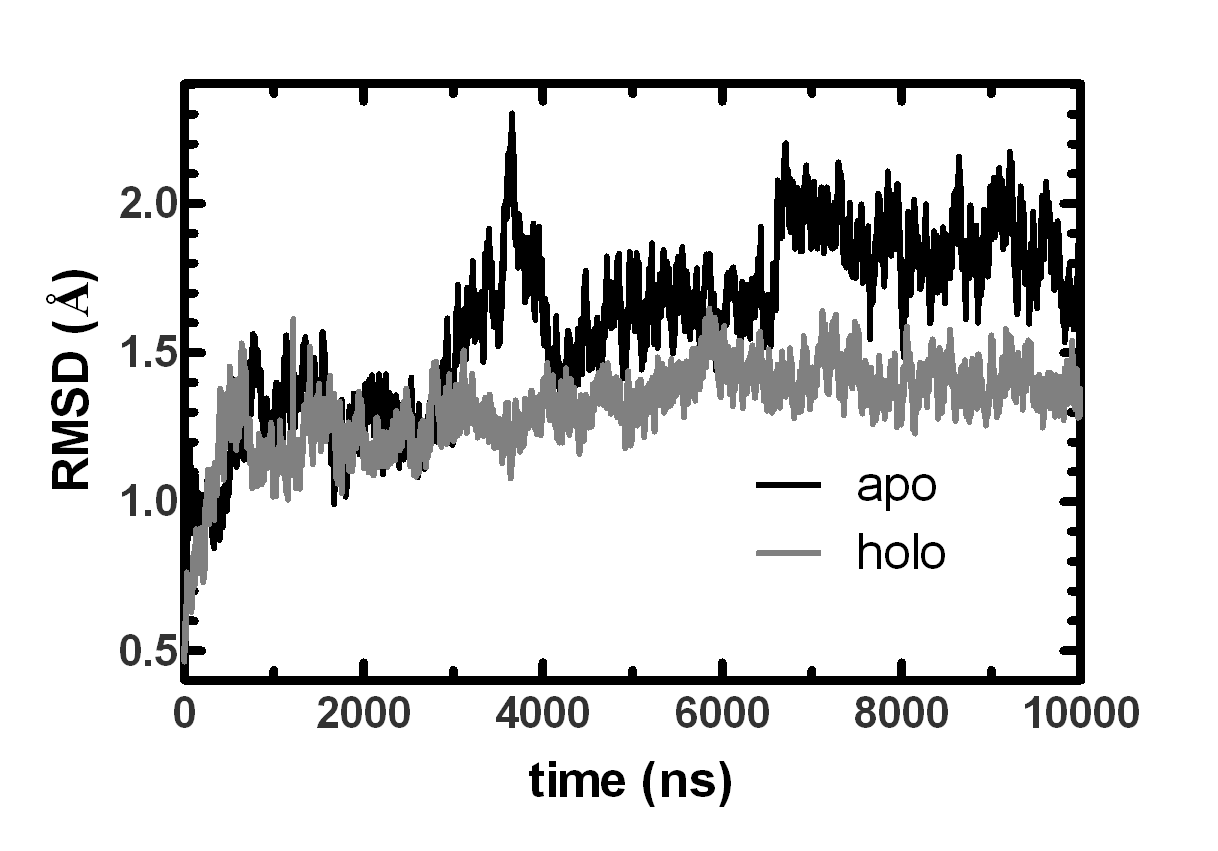

Supplement: Figure S1 — The RMSD values of the 10 ns long MD trajectories. The holo form is stabilized by the ligand, while the apo form displays larger fluctuations. (0.10 MB DOC) [file pcbi.1000544.s001.tif]
