# Supplementary material for: Perturbation-Response Scanning Reveals Ligand Entry-Exit Mechanisms of Ferric Binding Protein
Source: PLoS Comput Biol. 2009 Oct 23;5(10):e1000544. doi: 10.1371/journal.pcbi.1000544 (PMC2758672; doi:10.1371/journal.pcbi.1000544)
Supplement: Text S1 — Sample applications of the PRS method. (1.45 MB DOC) [file pcbi.1000544.s002.doc]

**S1. Sample applications of the PRS method**

In this section we provide results from the PRS method on different protein systems. Each example illustrates a different situation linked to mechanical changes in the system.

**A. Stabilization of Closed Conformation by Ligand Binding: K- R- Orn- Binding Protein**

The periplasmic lysine/arginine/ornithine binding protein (LAO) is a 238 residue long chain that contains two lobes moving relative to each other upon binding the substrate. Its apo- and holo (lysine bound) structures have been determined at 1.8 and 1.9 Å resolution, respectively, by x-ray crystallography [1]. It is one of the earlier examples whereby it was claimed that the role of the ligand is to stabilize the closed conformation upon binding, as opposed to the previous view of a direct conformational change induced by the ligand.

We have applied the PRS method to both the apo and the holo forms of this protein (PDB codes 2lao and 1lst, respectively; the overall RMSD between the two structures is 4.7 Å; *r_c_* = 9 Å). The results are summarized in Figure S1. In the apo form, a large number of residues in different parts of the protein yield high correlations (*C_i_* in equation 19) with the displacements obtained from x-ray structures (102 residues have *C_i_* ≥ 0.8). The holo form, on the other hand, is stabilized by the ligand, and perturbing only those residues in close proximity to the ligand (shown in orange in the figure) lead to higher *C_i_* values. Unlike FBP, long range contacts whose perturbations manipulate the ligand-binding region are not detected in LAO.


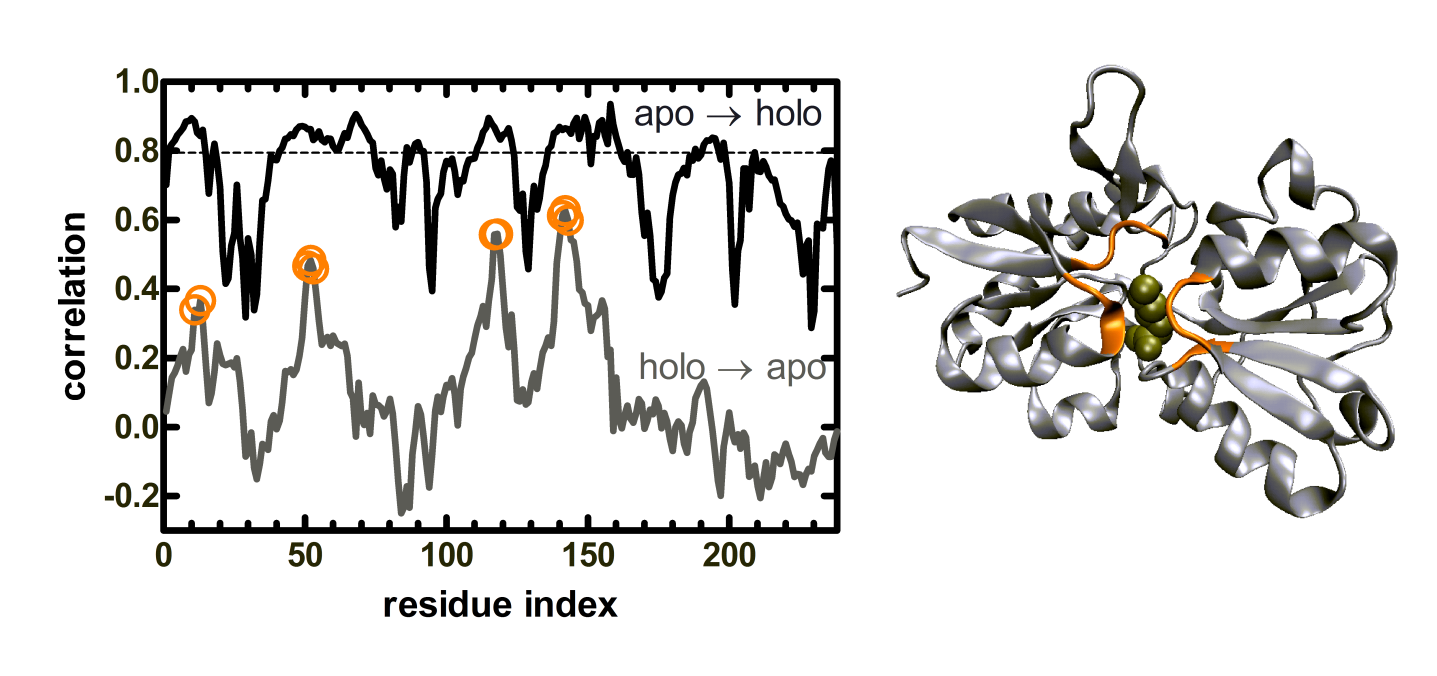


**Figure A.** **(Left panel)** The correlation coefficient between the displacement profiles from PRS and x-ray structures, for the apo (black curve), and the holo form (gray curve) as the initial structure; this is the counterpart of figures 4a and 4b for LAO. **(Right panel)** The x-ray structure of the holo form; the ligand lysine is colored in olive. The residues whose perturbation leads to higher correlations in the holo form are shown in orange in both panels, and all belong to the ligand binding site.

**B. Allosteric Control in CheY**

The chemotaxis protein Y (CheY) is a response regulator in two-component signal transduction systems. Its apo and holo (Mg^+2^ ion bound) structures have been determined at 1.7 and 1.8 Å resolution, respectively [2,3]. As a simple example of protein allostery, the activation mechanism in CheY was studied by molecular simulation [4]. Therein, it was proposed that the β4 – α4 loop acts as a gating element that remotely regulates the population of the active loop configuration.

We have studied the apo-form of CheY with PRS methodology (PDB code of apo and holo structures are 3chy and 1chn, respectively; the overall RMSD between the two structures is 1.4 Å; *r_c_* = 8 Å). The results are displayed in figure S2. The Mg^+2^ ion in the holo form coordinates residues 13, 57 and 59 (highlighted in olive in figure S2) along with three water molecules. The β4 – α4 loop is highlighted in orange. In conformity with the above-mentioned allosteric role of this loop, we find that forces exerted on this flexible region induce displacements on the order of 1 Å around the binding site.


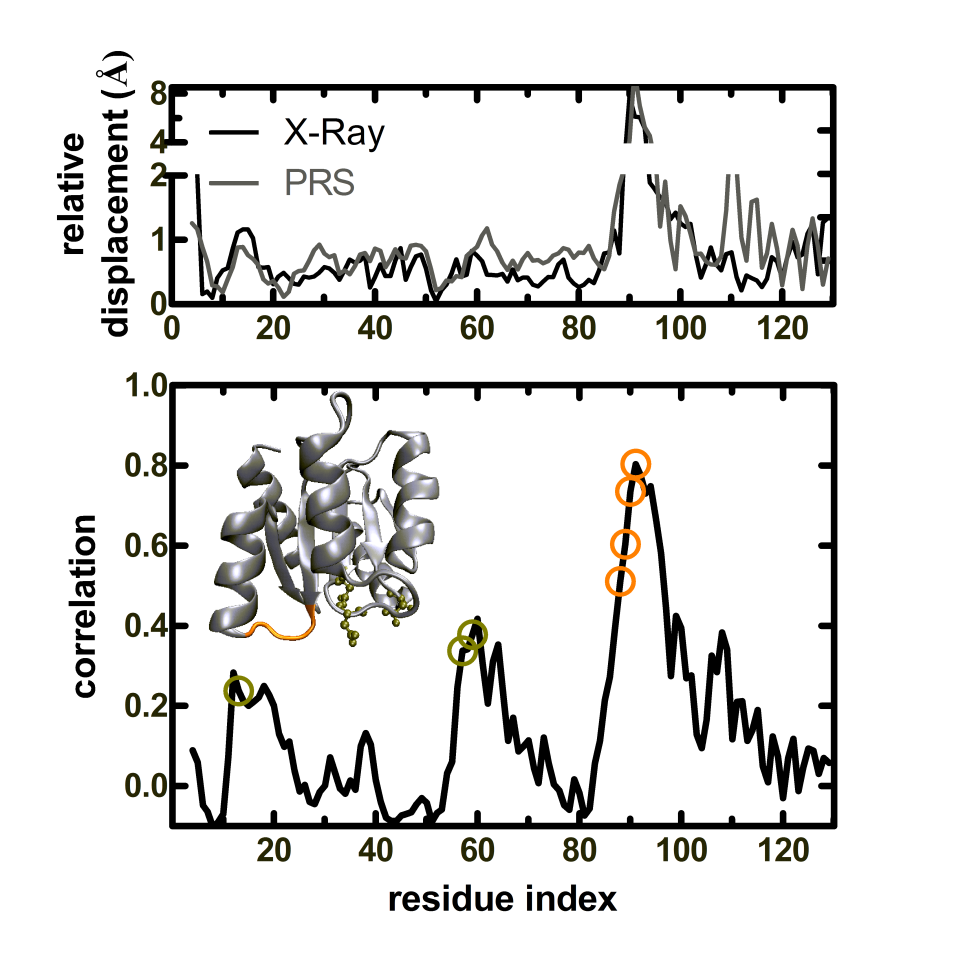
**Figure B.** **(Bottom panel)** The correlation coefficient between the displacement profiles from PRS and x-ray structures. The CheY apo-structure is shown in the inset. The residues directly contacting the Mg^+2^ ion in the bound form and the β4 – α4 loop residues are highlighted in the colors olive and orange, respectively. **(Top panel)** A sample displacement profile, as a result of a perturbation on residue 91 which resides in the β4 – α4 loop is exhibited. The displacements from the x-ray coordinates are also shown for comparison; residue 91 gives the highest displacement similarity to those recorded in the x-ray structures (the peak in the bottom panel curve).

**C. Open ↔ Closed Form Conformational Change in Bacteriorhodopsin in the Absence of Illumination:**

Bacteriorhodopsin functions as a light-driven proton pump residing in the cell membrane of *halobacterium salinarum*. It contains the chromophore retinal, which isomerizes to 13-cis retinal by absorbing a photon. This is followed by protein conformational changes occurring in the dark, resulting in the translocation of one or more protons through the membrane [5]. It was shown by electron microscopy and later confirmed by x-ray crystallography that, even in the absence of illumination, selected mutations could be used to partially destabilize the native conformation towards the open conformation [6,7]. In the extreme, it has even been possible to obtain the full conformational change using the triple mutant D96G/F171C/F219L, without any further significant changes upon illumination.

We have scanned the native conformation of bacteriorhodopsin to understand the extent to which singly inserted perturbations on the structure may drive it towards the open form. The results are displayed in figure S3 (PDB code of native and open conformations are 1fbb and 1fbk, respectively; the overall RMSD between the two structures is 0.95 Å; *r_c_* = 9 Å). We find that several residue residing on the cytoplasmic side of the **F** and **G** helices may be perturbed to yield average correlations of 0.4 – 0.6 with the experimentally determined conformational change. In particular, perturbations on residue T170 lead to the average correlation of ca. 0.6. Note that two of the residues in the experimentally determined triple mutant reside in the cytoplasmic side of helices **F** and **G** [8].


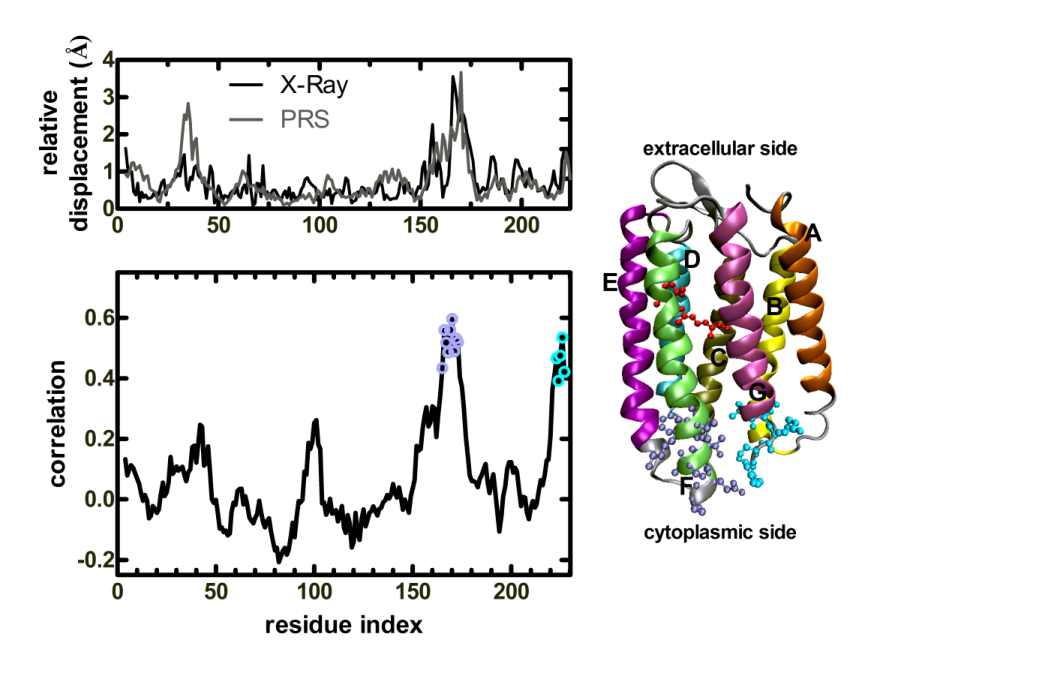
**Figure C.** **(Bottom left panel)** The correlation coefficient between the displacement profiles of the native and open conformations of bacteriorhodopsin from PRS and experimental structures. The residues yielding the highest correlations with the open conformation reside in the cytoplasmic side **F** and **G** helices, and are colored in purple and cyan, respectively. **(Top left panel)** A displacement profile, as a result of a perturbation on T170 which resides in the **F** helix. The displacements from the x-ray coordinates are also shown for comparison; this residue gives the highest displacement similarity to those recorded in the x-ray structures. **(Right panel)** The native conformation. The seven transmembrane helices are each colored differently and labeled. Retinal is shown in red ball-and-stick representation. Residues leading to the largest correlation with the experimental overall displacements upon singly inserted perturbations are colored in purple and cyan, in accord with the curve in the bottom left panel.

**D. Binding affinity of an antibody to different epitopes.**

In this example, we use the PRS methodology for a different purpose, namely, to differentiate the binding affinities of different ligands to the same protein. This is based on the argument that we expect tighter binding to be associated with inducing larger correlated motions in the overall protein. To test this idea, we use the interesting example of promiscuity in the primary immune response, that has been presented on the germline antibody 36-65 [9]. In this system, four distinct conformations are displayed in the unliganded state; yet, a common one of these is stabilized upon complexation with three different dodecapeptides. Interestingly, the positions of the peptides are different in the combining site, sharing only two Tyr residues. The structure of this common conformation is shown in figure S4, and the two paratope Tyr residues (Y50 and Y106) that reside in the variable domain are shown in space filling representation.

We have scanned this common conformation (PDB code 2a6j) using the PRS method (*r_c_* = 11 Å); results are shown in figure S4. The three peptides whose bound states are studied are **RLL**IADPPSPRE, **KLA**SIPTHTSPL and **SLG**DNLTNHNLR (PDB codes 2a6d, 2a6i and 2a6k, respectively), and are labeled by the first three residues. The RMSD between the unbound and bound structures of the variable domain (residues 1-128) are 0.98, 1.1 and 1.1 Å, respectively. Since the structural differences in the constant domain are very small, the forces inserted on residues in that region do not yield discriminating results. More interesting findings are obtained by perturbing the variable domain residues, whereby the degree of achieving higher correlations is in the order SLG > RLL > KLA; this is in accord with the measured [10] binding affinities of these peptides (0.21, 0.15 and 0.12 μM, respectively).


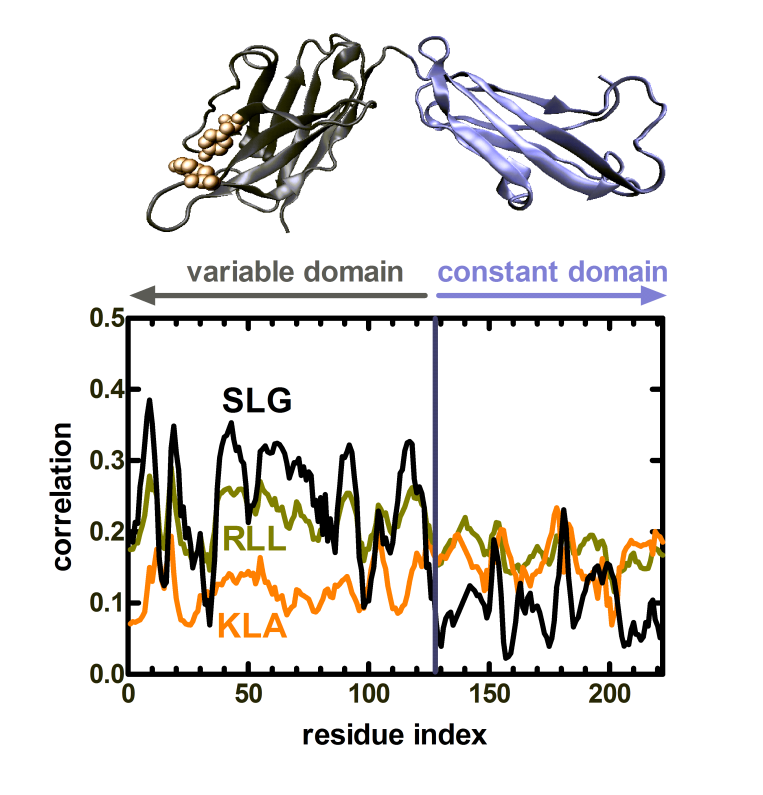
**Figure D.** **(Top panel)** The x-ray structure of the common peptide-bound conformation of the antibody heavy (H) chain. The variable (containing V_H_ and C_H1_) and constant domain residues (containing C_H2_ and C_H3_) are colored in gray and purple, respectively. **(Bottom panel)** The correlation coefficient between the PRS and x-ray structure displacement profiles of the unbound and bound structures for the three peptides. The higher correlations obtained by perturbing the variable domain residues are in accord with the binding affinities of the peptides.

**E. N-lobe of human transferrin**

As discussed at length in the Introduction, human transferrin (hT) has many structural features common to bacterial FBP. The three-dimensional structure of only the apo form of full length hT is available. However, the x-ray structures of the N-lobe of apo- and holo-hT have been determined to 1.6 and 2.2 Å, respectively (PDB codes 1BP5 [11] and 1A8E [12]). Sequence identity of N-lobe hT with FBP is 12 % and sequence similarity is ca. 62 %; hT has 337 residues as opposed to FBP having 309. Structural alignment using MultiProt [13] of bacterial FBP and human transferrin N-lobe, both in the Fe bound form, yields 187 residues aligned at an RMSD of 3.48 Å. Although the overall structure with the two domains enclosing the Fe ion persists in N-lobe hT with many of the secondary structural elements having corresponding locations, overall there are structural shifts as well as loop insertions in hT. In particular, the Fe ion is located deeper in the groove between the two domains. As a result, there are 16 residues whose C_α_ atoms reside within 8 Å of the ion. Six of these (L62, D63, A64, G65, S248, H249) are located in the moving domain and the others (R124, S125, A126, G127, Y188, V205, K206, V246) are in the fixed domain. The structure of holo-hT is shown in the top panel of Figure S5, and the residues in contact with Fe ion are marked on the figure.

By applying PRS on the apo form (using *r_c_* = 8 Å as in FBP), we find that 60 % of the residues yield correlations better than 0.9. However, only five residues (A10, N183, K278, A299, H300) yield such good correlations when the holo form is perturbed. These results are shown in orange on Figure S5. Of these five residues, K278, which is 25 Å away from the ion, is found to organize the moving domain residues in contact with the ligand, as shown in the inset to the bottom panel of Figure S5. All six residues move parallel to each other when K278 is perturbed, with the angle *θ* between any two pairs (equation 21) being in the interval 1 – 20°. Moreover, the response is collected along a line on all the six residues (i.e. the fractional contribution of the highest eigenvalue *p*_1_ of the response sub-matrix (equation 20) is in the range 0.68 – 0.81 for these residues). Such a collectivity, along with the correct displacement profile of the residues, is obtained only when this distant residue is perturbed. Note that K278 has also been identified as an allosteric kinetically significant anion binding site in MD/molecular mechanics simulations in the presence of Cl^-^ [14].


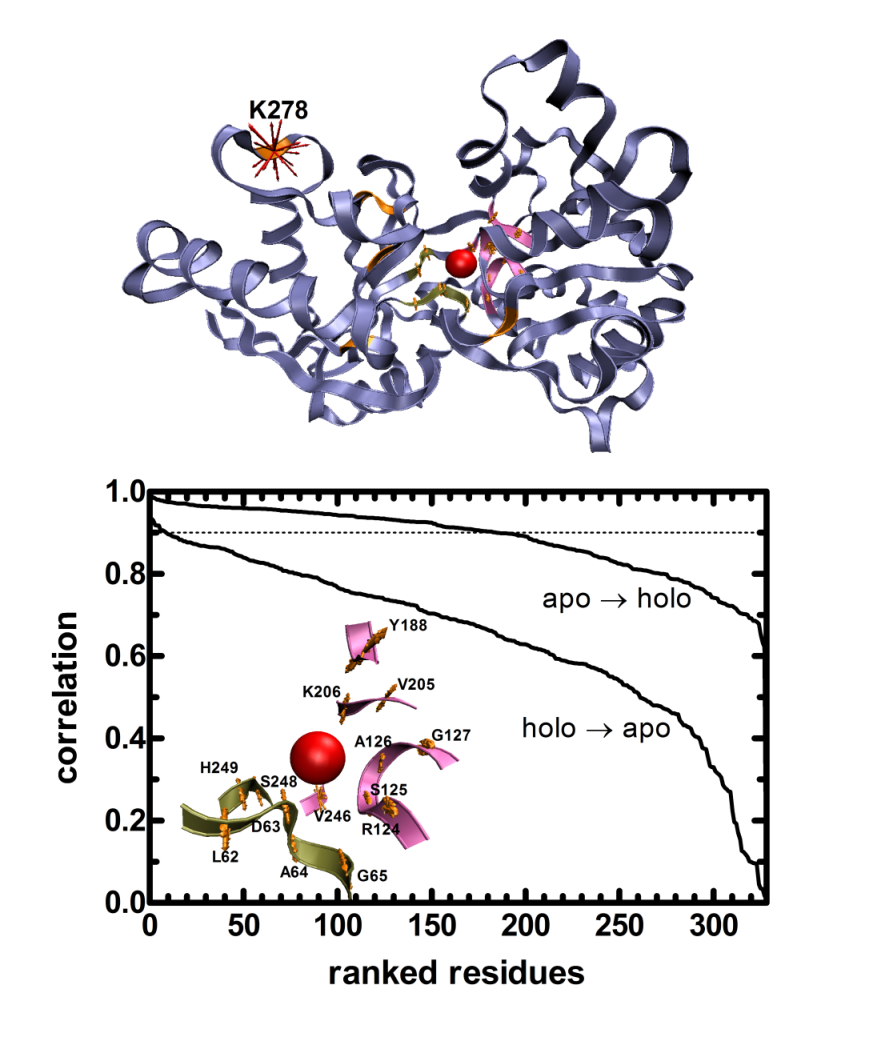
**Figure E.** **(Top)** The x-ray structure of the N-lobe of hT, holo form. Fe ion is shown in red. Residues whose C_α_ atoms are within 8 Å of the ion are shown in green (moving domain) and pink (fixed domain), respectively. **(Bottom)** The correlation coefficient between the displacement profiles from PRS and x-ray structures, for the apo (upper curve), and the holo form (lower curve) as the initial structure, sorted from largest to smallest value (counterpart of figure 4c for hT). Residues A10, N183, K278, A299, H300 (marked in orange on the x-ray structure) give the highest correlation (*C_i_* > 0.9) in the holo form; conversely, 60 % of the perturbations lead to the large correlations in the apo form. **(Inset)** Magnified response of the Fe-contacting residues to random perturbations on K278 induce a correlated response in the moving domain residues, all six having a preferred direction (green), whereas the displacements in the fixed domain residues (pink) are uncorrelated.

**References**

1. Oh BH, Pandit J, Kang CH, Nikaido K, Gokcen S, et al. (1993) 3-Dimensional Structures of the Periplasmic Lysine Arginine Ornithine-Binding Protein with and without a Ligand. Journal of Biological Chemistry 268: 11348-11355.

2. Bellsolell L, Prieto J, Serrano L, Coll M (1994) Magnesium Binding to the Bacterial Chemotaxis Protein Chey Results in Large Conformational-Changes Involving Its Functional Surface. Journal of Molecular Biology 238: 489-495.

3. Volz K, Matsumura P (1991) Crystal-Structure of Escherichia-Coli Chey Refined at 1.7-Å Resolution. Journal of Biological Chemistry 266: 15511-15519.

4. Formaneck MS, Ma L, Cui Q (2006) Reconciling the "old" and "new" views of protein allostery: A molecular simulation study of chemotaxis Y protein (CheY). Proteins-Structure Function and Bioinformatics 63: 846-867.

5. Kaulen AD (2000) Electrogenic processes and protein conformational changes accompanying the bacteriorhodopsin photocycle. Biochimica Et Biophysica Acta-Bioenergetics 1460: 204-219.

6. Hirai T, Subramaniam S (2009) Protein Conformational Changes in the Bacteriorhodopsin Photocycle: Comparison of Findings from Electron and X-Ray Crystallographic Analyses. PLoS One 4: e5769.

7. Subramaniam S, Lindahl I, Bullough P, Faruqi AR, Tittor J, et al. (1999) Protein conformational changes in the bacteriorhodopsin photocycle. Journal of Molecular Biology 287: 145-161.

8. Subramaniam S, Hirai T, Henderson R (2002) From structure to mechanism: electron crystallographic studies of bacteriorhodopsin. Philosophical Transactions of the Royal Society of London Series a-Mathematical Physical and Engineering Sciences 360: 859-874.

9. Sethi DK, Agarwal A, Manivel V, Rao KVS, Salunke DM (2006) Differential epitope positioning within the germline antibody paratope enhances promiscuity in the primary immune response. Immunity 24: 429-438.

10. Manivel V, Bayiroglu F, Siddiqui Z, Salunke DM, Rao KVS (2002) The primary antibody repertoire represents a linked network of degenerate antigen specificities. Journal of Immunology 169: 888-897.

11. Jeffrey PD, Bewley MC, MacGillivray RTA, Mason AB, Woodworth RC, et al. (1998) Ligand-induced conformational change in transferrins: Crystal structure of the open form of the N-terminal half-molecule of human transferrin. Biochemistry 37: 13978-13986.

12. MacGillivray RTA, Moore SA, Chen J, Anderson BF, Baker H, et al. (1998) Two high-resolution crystal structures of the recombinant N-lobe of human transferrin reveal a structural change implicated in iron release. Biochemistry 37: 7919-7928.

13. Shatsky M, Nussinov R, Wolfson HJ (2004) A method for simultaneous alignment of multiple protein structures. Proteins-Structure Function and Bioinformatics 56: 143-156.

14. Amin EA, Harris WR, Welsh WJ (2004) Identification of possible kinetically significant anion-binding sites in human serum transferrin using molecular modeling strategies. Biopolymers 73: 205-215.
